# The Common Task

**Published:** 1907-10-05

**Authors:** 


### THE COMMON TASK.

Correspondence and Queries for this section should be sent to the Editor of The Hospital, 28 Southampton Street, Strand, London, and marked "Nursing Administration."

### The Institution Cook.

Few people realise the importance of this functionary. Through his or her hands pass every year in a large hospital many thousand pounds' worth of provisions; the economical administration of the household largely depends on the way in which these articles are dealt with in the kitchen. The successes of a good housekeeper are achieved more than is ever known by means of co-operation with a good cook. It is unfortunate, then, that the position of the cook in big institutions is still an entirely subordinate one, and that her position as the head of one of the most important administrative departments is not differentiated from that of the other servants. As a matter of fact, too often she is merely another servant. Her training has given her no knowledge of the art of economy, and her skill is limited to the best manner of heating her ovens and regulating the boilers. She can deal with big joints which require muscle for their manipulation, but the inner mysteries of food preparation and flavourings are a sealed book to her. It may be urged that this is the fault of the national incapacity for cookery; or that good cooks are not to be attracted to institution work. Neither of these two reasons is adequate to account for the general incapacity of the institution cook. The real fact is that the wages offered are not sufficient to secure the man or woman who would be capable of ruling the kitchen in the interests of the charity. The usual wages given run from £35 to £60 a year, and even the higher figure is not adequate when the heavy work and responsibilities of the position are taken into account, together with the long and expensive training which is necessary to qualify for it. In one hospital at least in London the experiment of engaging a good chef has been tried. The salary paid is £120 a year. The result is in every way a complete success. The cost of the board for the staff works out under this régime at 6s. a week per head, and the comfort of staff and patients alike is in striking contrast to the hardships endured in many quarters where nearly double this amount is muddled away. The manner in which delicious dishes are prepared, as if by magic out of the most elementary ingredients, is a continual source of astonishment to the housekeeper. It is, of course, the cook's business to think out ways of dealing with the materials at hand and prepare the menu, not that of the overburdened housekeeper, into whose training this branch of work cannot reasonably be expected to enter. For the extra £60 or £70 a year expended on the cook's salary, the institution reaps a rich harvest of comfort and well-being through the mere fact that the staff is well fed, and if the saving be expressed in money it cannot be less than £1,000 a year, reckoning that at least a shilling a head a week is saved by firm administration in the kitchen.

### The Four Years' Course.

At the root of the question whether a four years' course should be introduced into hospitals, lies the question of the proper proportion of first-year probationers for the hospital to have in training. The proportion which now prevails is from 25 to 28 per cent., and in the judgment of many it is this large number of unskilled workers which constitutes the strain in modern hospital work. It is not only that the newcomers are of comparatively small use for the first few months, it is that much of the time of the skilled workers is taken up in teaching them. If the course were expanded to four years it is obvious that the number of probationers in training would be proportionately reduced. Every hundred nurses would then be made up somewhat as follows:—Superintendents and sisters, 20; staff nurses, fourth year, 20; probationers, third year, 20; probationers, second year, 20; probationers, first year, 20. There is little doubt that such a redistribution of the staff would render the work more regular, and avoid much of the tense pressure which is apt to wear out the matron before her time. But the question cannot be settled off hand on its own merits. Are we prepared to reduce the number of nurses at present in training by 15 per cent.? It does not appear that the number of nurses turned out yearly by the training schools is at all in excess of public requirements, and a shortage of trained nurses would sooner or later make itself felt were the number of pupils curtailed.

---

THE HOSPITAL *October* 5, 1907.

